# Correlation of non-suicidal self-injury with SLC6A4 promoter DNA methylation in children and adolescents with bipolar depression

**DOI:** 10.3389/fpsyt.2026.1773114

**Published:** 2026-05-19

**Authors:** Alimire Alimujiang, XiaoQin Shen, ChengJi Wang, ShaoHong Zou

**Affiliations:** 1 Graduate School, Xinjiang Medical University, Urumqi, Xinjiang Uygur Autonomous Region, China; 2 Department of Clinical Psychology, People’s Hospital of Xinjiang Uygur Autonomous Region, Urumqi, Xinjiang Uygur Autonomous Region, China

**Keywords:** bipolar disorder, children and adolescents, gene methylation, non-suicidal self-injury, SLC6A4

## Abstract

**Purpose:**

To investigate the association between non-suicidal self-injury (NSSI) and DNA methylation of the SLC6A4 promoter region in individuals diagnosed with bipolar depression.

**Patients and methods:**

A total of 48 children and adolescent patients meeting ICD-10 criteria for bipolar disorder with current depressive episodes (Hamilton Depression Rating Scale-24 score >20) were recruited, and stratified into two groups according to the presence or absence of non-suicidal self-injurious (NSSI) behavior(NSSI group and non-NSSI group). Negative life events were evaluated using the Adolescent Self-Rating Life Events Checklist (ASLEC). The methylation levels of five CpG sites within the SLC6A4 promoter region were determined by pyrosequencing, and mRNA expression was assessed by quantitative reverse transcription polymerase chain reaction (qRT-PCR).

**Results:**

In 48 adolescents with bipolar depression, the two groups were comparable in age, gender, education, only-child status, family history of mental illness, and disease course (all *p* > 0.05). Compared with the non-NSSI group, the NSSI group showed significantly lower CpG2 methylation at the SLC6A4 promoter, higher SLC6A4 mRNA expression, and higher ASLEC scores. The other four CpG sites showed no significant differences. Spearman’s correlation revealed a strong negative association between CpG2 methylation and mRNA expression (*ρ* = -0.547, *p* < 0.001). Two logistic regression models both fit the data well. Higher CpG2 methylation was a protective factor against NSSI (OR = 0.131, *p* = 0.021), while higher mRNA expression was a risk factor (OR = 10.957, *p* = 0.015). ASLEC scores and interaction terms were not significant.

**Conclusion:**

These preliminary findings suggest that lower CpG2 methylation and higher SLC6A4 mRNA expression are each independently associated with NSSI in adolescents with bipolar depression. Stressful life events were higher in the NSSI group, but did not significantly moderate these associations in the current sample. Given the small sample size and cross-sectional design, the results should be viewed as exploratory and require replication in larger studies.

## 1 Introduction

Bipolar disorder (BD) is a highly widespread mental health condition that is linked to a significantly elevated mortality risk (1). A systematic review indicates that bipolar disorder most often begins in early life, with an average age of 17 (2). Between 1990 and 2017, the incidence of BD rose by 47.74% worldwide, with disability-adjusted life years rising by 54.4%. Notably, individuals aged 10 to 19 years accounted for the largest proportion of cases (3). Non-suicidal self-injury (NSSI) is a prominent feature of bipolar disorder, and its rapidly increasing prevalence underscores the need for heightened clinical attention. The DSM-5 characterizes NSSI as exhibiting deliberate, intentional, and repetitive infliction of harm on one’s own body without any suicidal intent and without resulting in death. This behavior is particularly prevalent among children and adolescents. According to the study, patients with BD who exhibited NSSI developed the condition earlier than those who did not (4). The lifetime incidence of NSSI in children and adolescents is still high, according to a scoping review of the topic in the Chinese population. It is predicted to be 29.3% among primary school students, 25.3% among middle school students, and 32.8% among high school students (5). It has been discovered that 25–35% of teenagers in random samples taken from German schools have experienced at least one episode of NSSI. The prevalence of NSSI in clinical populations has been reported to be as high as 50% (6). Individuals with pediatric and adolescent bipolar disorder often exhibit more severe symptoms and face a more adverse prognosis compared to adults with BD. This condition is associated with increased risks of relationship difficulties, academic failure, and elevated suicide rates (7).

The development of NSSI is influenced by genetic factors, early-life stress, and stress-regulation disorders (8). The SLC6A4 gene is a focal point in studies examining the correlation between epigenetic mechanisms and NSSI behavior. The SLC6A4 gene, located on the long arm of chromosome 17 (17q11–17q12), spans spans approximately 35 kilobases (kb) and comprises 13–14 exons. This gene encodes 5-HTT and is regulated by biallelic polymorphisms in the promoter region, specifically the serotonin transporter-linked polymorphic region (5-HTTLPR), situated upstream of the transcription start site (9). A study conducted using a non-human primate model to investigate human NSSI behavior demonstrated that the SLC6A4 genotype influences distinct NSSI behaviors. Specifically, subjects homozygous for the L allele exhibited elevated plasma adrenocorticotropic hormone levels and pronounced stress-induced stereotypic behaviors. In contrast, subjects carrying the S allele displayed more impulsive (10) behaviors. Dell’osso et al.’s research (11) on genetic predictors of suicidal ideation and self-harm identified a significant relationship between the SS genotype and self-harming behaviors. Additionally, studies examining the connection between SLC6A4 polymorphisms and borderline personality disorder revealed that SLC6A4 genetic variations might be linked to BPD, which is frequently associated with NSSI and impulsive aggression (12). Hankin et al.’s investigation (13) on stress-environment interactions suggested that adolescents carrying at least one short allele of the 5-HTTLPR polymorphism who experienced interpersonal stress were at higher risk for developing NSSI behaviors. Self-directed aggression (includes suicidal behavior, self-mutilation) was more common among carriers of 5-HTTLPR high (LALA), intermediate (LALG, SLA) activity variants than in carriers of the low activity (LGLG, SLG, SS) variant, according to the study about investigating predictors of self-aggression among Italian prisoners (14). Neurobehavioral outcomes may be influenced by interactions between the 5-HTTLPR and early stress, potentially involving epigenetic alterations. 5-HTT expression is controlled by methylation of the CpG island in the 5-HTT LPR; in human lymphoblast cell lines, higher average DNA methylation is associated with lower 5-HTT expression (15).

Based on previous studies, SLC6A4 gene polymorphisms have been associated with NSSI. DNA methylation in the SLC6A4 promoter has been shown in previous experimental studies to regulate its gene expression, which is involved in 5-HT signaling and emotional processing. Research exploring the relationship between SLC6A4 promoter methylation and NSSI among children and adolescents with bipolar depression remains notably limited in the current literature. Our cross-sectional investigation employed pyrosequencing to quantify DNA methylation levels in the SLC6A4 promoter region and to conduct an initial examination of how these methylation patterns correlate with NSSI behaviors in this specific population. These preliminary observations offer valuable groundwork that may inform and guide more comprehensive investigations in the future.

## 2 Materials and methods

### 2.1 Participants

This research employed a case-control design, utilizing both a general questionnaire and psychometric scales. Regardless of gender, 48 patients with bipolar depression were included in this study. These patients received treatment at the Department of Clinical Psychology, People’s Hospital of the Xinjiang Uygur Autonomous Region, from June 2022 to December 2023.

The study received approval from the Ethics Committee of the People’s Hospital of the Xinjiang Uygur Autonomous Region (Ethical Opinion No.KY2023060127). All participants, along with their families, provided informed consent after being fully briefed on the study’s objectives.

The criteria for study enrollment were as follows (1): Diagnosis of BD according to the ICD-10 criteria, confirmed by two qualified psychiatric physicians (2); A Hamilton Rating Scale for Depression 24 (HRSD24) score > 20 points (3); Age between 12~18 years old, regardless of gender (4); No treatment received for at least one month before admission (5); Written informed consent from participants willing to participate in the study; and (6) Ability to cooperate with the completion of questionnaire evaluations and the collection of related gene methylation blood samples. The exclusion criteria are as follows (1): Other mental disorders or intellectual disabilities (2); History of head trauma, brain organic diseases, epilepsy, or other neurological conditions; serious medical conditions, particularly endocrine or rheumatic immune disorders associated with mood changes (3); Previous use or dependence on psychotropic substances (4); A score of 2 or above on item 3 (suicidal ideation) of the Hamilton Depression Rating Scale 24, where two points indicate persistent thoughts about death or a wish to die; and (5) Alcohol or drug abuse within the past three months.

### 2.2 Scales assessment

Non-suicidal self-injury was evaluated using the Adolescent NSSI Assessment Questionnaire (16). The behavioral questionnaire comprises 12 items. NSSI is identified in individuals who exhibit at least one of the 12 common forms of self-injury outlined in the questionnaire.

The stressful life events experienced by adolescents were assessed using the ASLEC. This self-report scale requires participants to indicate whether an event occurred within the past year and to rate its impact based on their psychological state at the time. Ratings range from no impact (1) to very severe impact (5). Statistical analysis focuses on two main aspects: the frequency of events and the overall stress level. Events that did not occur are recorded, and the cumulative score reflects the total stress experienced. Additionally, the data can be further analyzed by dividing it into six factors for detailed statistical evaluation.

### 2.3 Molecular analysis

#### 2.3.1 Materials and instruments

All experimental reagents/consumables and experimental instruments are listed in (**Tables 1**, **2**).

**Table 1:** Experimental reagents/consumables.

Name	Manufacturer
Solution-based DNA Extraction Kit	Junnuo De
Agarose	Shanghai Sangon
Nucleic Acid Stain	Shanghai Sangon
M5 HiClear DL2000 DNA Marker	JumboBio
Methylated DNA Bisulfite Conversion Kit	Zymo
Primer Synthesis	Thermo Fisher
EpiTYPER™ Reagent Kit	Agena, Inc
MassARRAY EpiTYPER™ Software	Agena, Inc

**Table 2:** Experimental instruments.

Name	Manufacturer
Nucleic Acid and Protein Quantifier	Hangzhou Aokang
Electrophoresis Power Supply	Beijing Liuyi Instrument Factory
Electrophoresis Tank	Beijing Liuyi Instrument Factory
Gel Imaging System	Shanghai Tianneng Technology Co., Ltd.
High-Speed Centrifuge	Eppendorf
Automatic Nucleic Acid Extractor	BioTeKe Corporation
NanoDrop2000	Thermo
Smart View Pro 1100 Gel Electrophoresis Imager	Major Science
MP-300V Electrophoresis Power Supply	Major Science
Large Electrophoresis Tank	Beijing Liuyi Biotechnology Co., Ltd.
ABI Veriti-96 PCR System	Applied Biosystems
ABI Veriti-384 PCR System	Applied Biosystems
384-Well SpectroCHIP® Bioarray Chip	Agena, Inc
MassARRAY Nanodispenser	Agena, Inc
MassARRAY Analyzer 4.0 Mass Spectrometer	Agena, Inc
Pipette	Eppendorf
qPCR 384-Well Plate	Applied Biosystems

#### 2.3.2 Target region selection and CpG island identification

**Figure 1:**
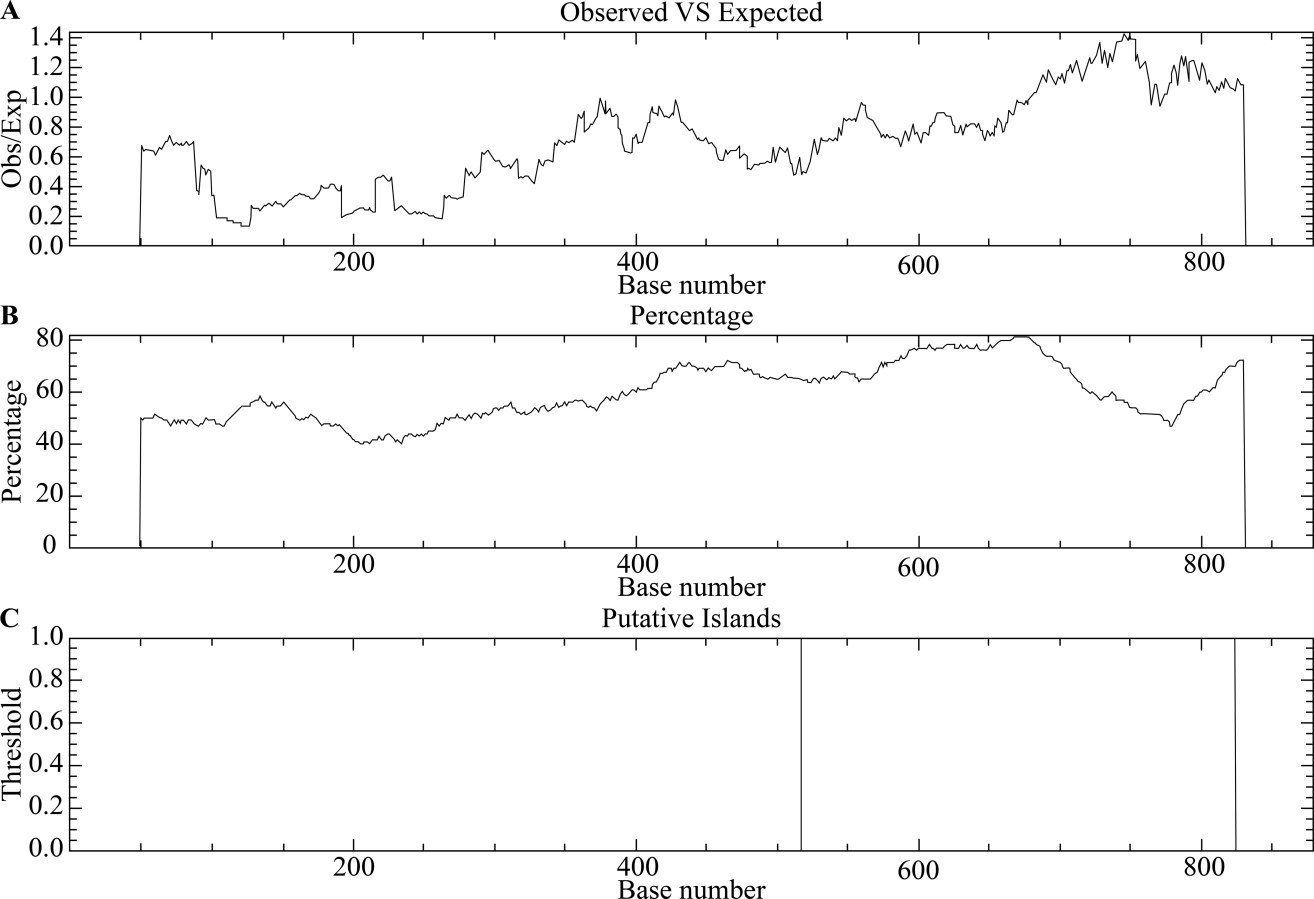
Prediction of putative CpG islands in the target sequence using EMBOSS CpGPlot. The analysis was performed with the following criteria: observed/expected (Obs/Exp) CpG ratio > 0.60, GC content > 50.00%, and minimum island length > 200 bp. **(A)** Observed/expected CpG ratio across the sequence; **(B)** GC content percentage across the sequence; **(C)** The identified CpG island, spanning positions 518–824 bp (length: 307 bp).

**Figure 2:**
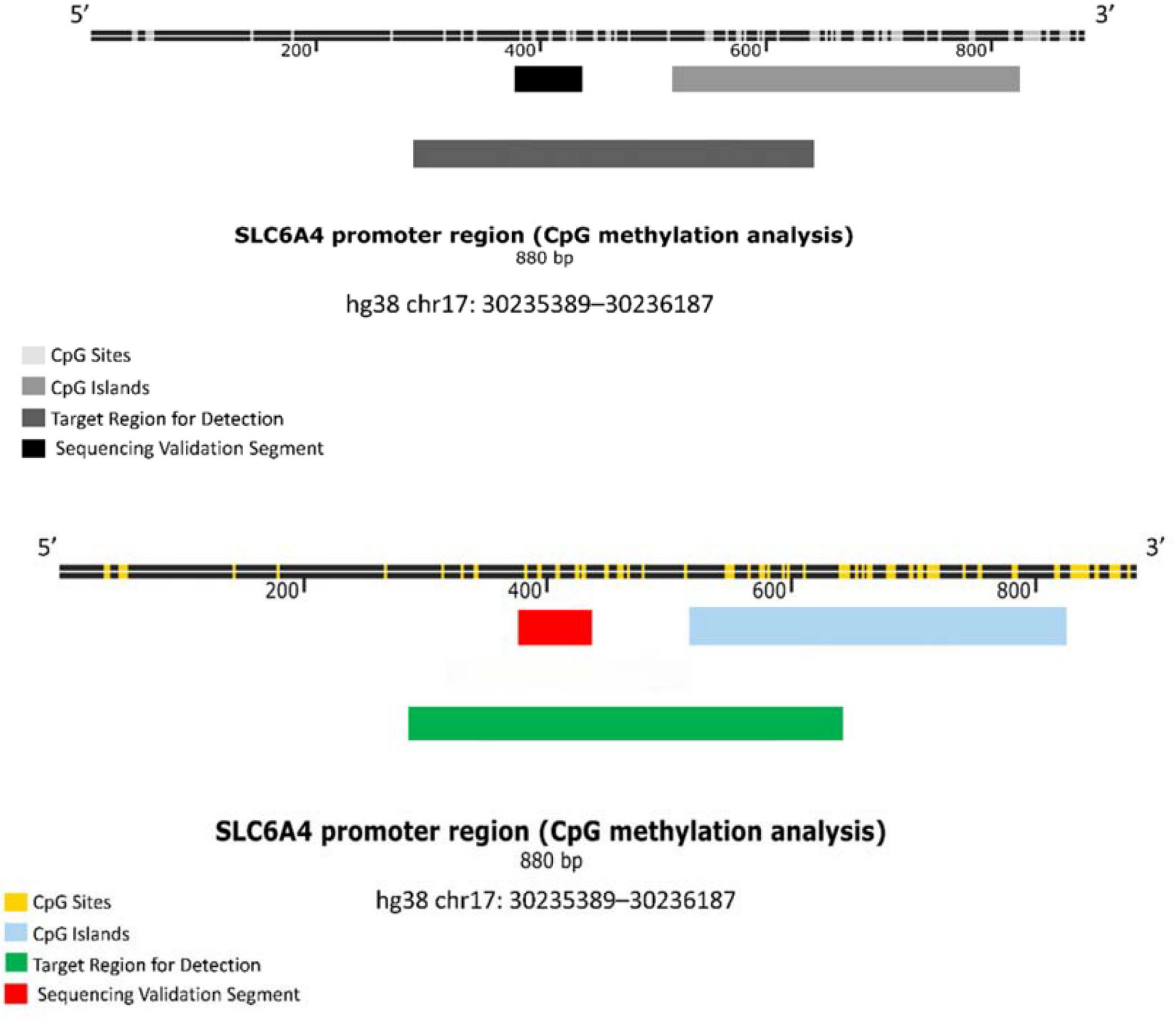
Genomic sequence. The 880-bp target region (hg38 chr17: 30235389–30236187) is shown. Yellow bars indicate CpG sites, light blue bars indicate CpG islands, the green bar denotes the target detection region, and the red bar marks the sequencing validation segment.

**Figure 3:**
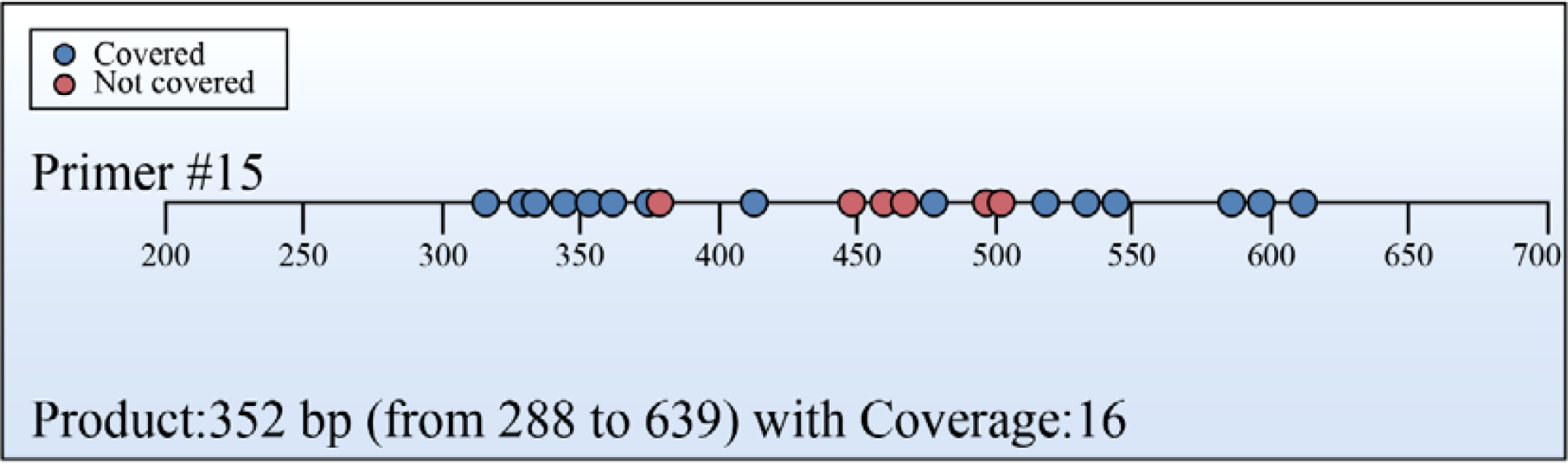
Sequence diagram. This figure details the amplification coverage (Product: 352 bp, 288–639 bp) of Primer #15 across the target region. Blue dots represent covered bases, while red dots indicate non-covered regions. The high coverage rate confirms the suitability of this primer for pyrosequencing.

The target gene sequence was analyzed using an online CpG island prediction tool, which identified one CpG island (**Figure 1**). The genomic sequence is presented in (**Figure 2**). A detailed sequence map is shown in (**Figure 3**), with dots representing CpG sites; blue-marked CpG sites were detectable, while red-marked sites were undetectable due to sequencing interference.

#### 2.3.3 Primer design

**Figure 4:**
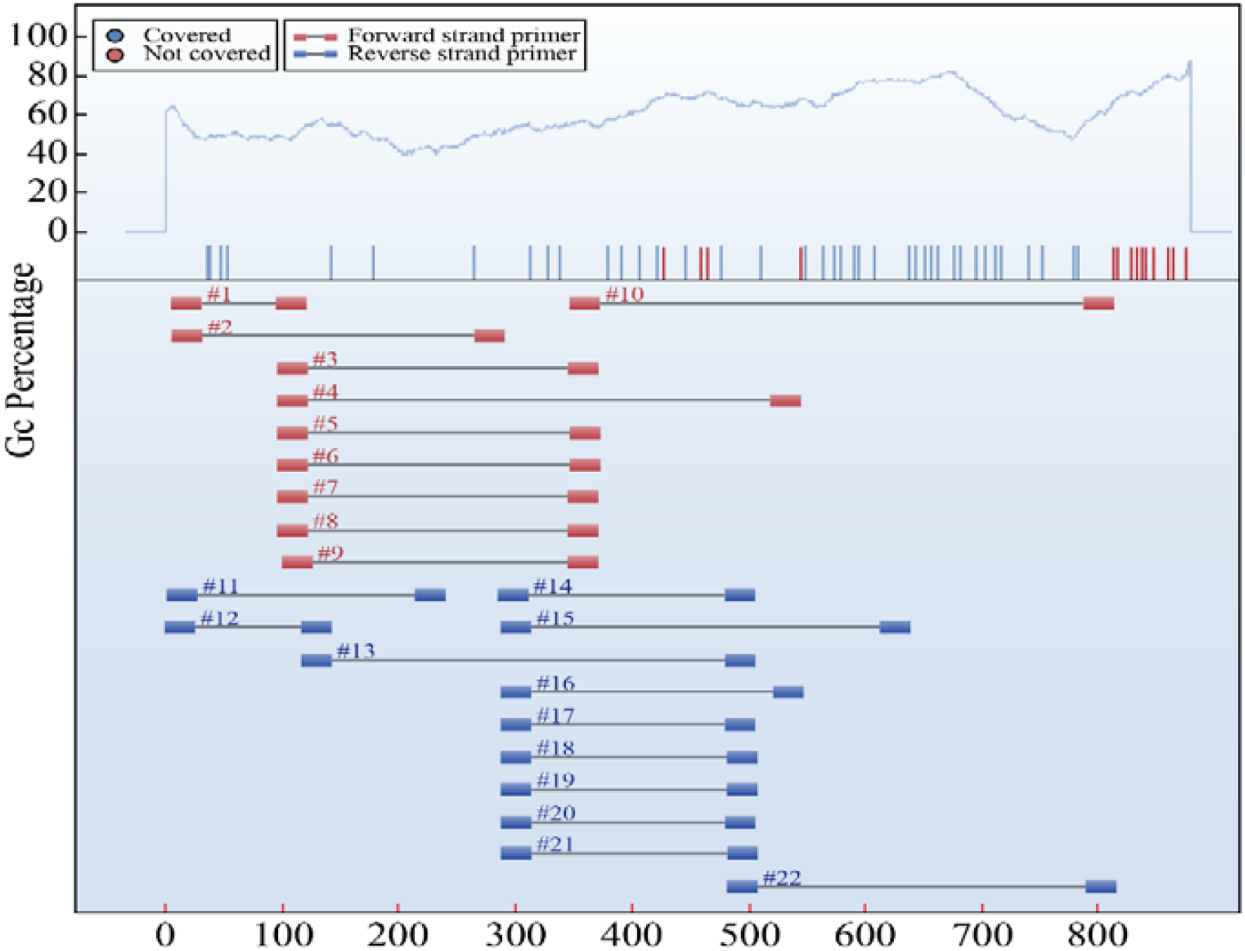
Primer design, amplicon coverage, and GC content analysis for SLC6A4. The upper panel displays the GC percentage profile (light blue line). The lower panel shows the amplicon ranges of all 22 designed primer pairs (red for forward, blue for reverse), validating the comprehensive coverage of the target region.

Primers were designed using the Agena EpiDesigner software. Primer design criteria were as follows: primer binding sites were required to contain at least four non-CpG cytosines to monitor bisulfite conversion efficiency, with a required conversion rate of ≥95%. Priority was given to CpG sites free of sequencing interference, while avoiding repetitive sequences and regions with potential secondary structures. All primers were purified by HPLC or PAGE purification. Synthesized primers were subjected to pre-amplification testing, followed by sequencing validation to confirm target coverage and signal intensity. Primer sequences are provided in (**Table 3**), primer design and target coverage are illustrated in (**Figure 4**).

**Table 3:** Primer sequences.

Primer	Start	Size	Tm	GC%	C's
5’-end primer	639	25	61.85	44	7
3’-end primer	288	25	59.95	40	4
PRODUCT Size: 352, No of CpG's :23, Coverage : 16					

The 3’-end primer is labeled with a T7 promoter tag and an 8-base insertion sequence; the 5’- end primer is added with a 10.base tag to balance the Tm values of the forward and reverse primers.

#### 2.3.4 DNA extraction

Fasting venous blood was collected from all participants in EDTA-anticoagulated tubes. DNA extraction was performed as follows: 200 μL of thawed anticoagulated blood was mixed with 600 μL of fresh 1× RBC lysis buffer, incubated at room temperature for 10 min (with mixing), and then centrifuged at 12,000 rpm for 1 min. The pellet was re-suspended in 300 μL of RBC lysis buffer, incubated for 2 min, re-centrifuged, and the supernatant was discarded. After lysis with 300 μL of preheated (50 °C) nuclear lysis buffer (gentle pipetting to avoid shearing) and incubation at 60–65 °C for 45 min until clear, 150 μL of protein precipitation solution was added, vortexed for 25 s, and centrifuged at 12,000 rpm for 5 min. Approximately 450 μL of supernatant was transferred, mixed with 450 μL of isopropanol (30 inversions) until DNA precipitated, and then centrifuged for 5 min; the supernatant was discarded. The pellet was rinsed twice with 500 μL of 70% ethanol (each centrifuged for 3 min). The residual ethanol was removed by brief centrifugation (30 s) and air-drying (1 min), and the DNA was dissolved in 100 μL of ddH_2_O. Concentration, purity (nucleic acid-protein quantifier), and integrity (agarose electrophoresis) were verified; qualified samples were stored at -20 °C.

#### 2.3.5 Bisulfite conversion

Bisulfite conversion of genomic DNA was carried out using the EpiTect Bisulfite Kit. For DNA conversion, the DNA was first dissolved with the sulfite reagent by adding 800 μL of RNase-free water to each sulfite mixture and mixing thoroughly. Relevant reagents were prepared in 200 μL thin-walled PCR tubes as specified in (**Table 4**). The tubes were capped, mixed thoroughly, and incubated at room temperature (15–25 °C). The reaction conditions for DNA conversion were set on a PCR instrument as outlined in (**Table 5**), with an approximate duration of 5 h. Following the conversion cycle, the PCR tubes were centrifuged, and the reaction solution was transferred to a 1.5 mL tube. Fresh Buffer BL (560 μL, containing 10 μg/mL carrier RNA) was added, vortexed, and briefly centrifuged. The mixture was loaded onto EpiTect spin columns, centrifuged at maximum speed for 1 min, and the filtrate was discarded. Buffer BW (500 μL) was added, centrifuged, and the filtrate discarded. Subsequently, 500 μL of Buffer BD was added, incubated at 15–25 °C for 15 min, centrifuged, and the filtrate discarded. The washing step with 500 μL of Buffer BW was repeated twice. The columns were then transferred to new 2 mL tubes and centrifuged for 1 min to remove residual liquid. To evaporate the remaining ethanol, the columns with open lids were placed in new 1.5 mL tubes and incubated at 56 °C for 5 min. Finally, the columns were placed in new 1.5 mL tubes, 20 μL of Buffer EB was applied directly to the membrane center, and purified DNA was eluted by centrifugation at maximum speed for 1 min.

**Table 4:** Sulfite modification reaction components.

Component	Volume (μl)
DNA solution (1–500 ng)	Variable (max 40)
RNase-free water	Variable
Dissolved sulfite mixture	85
DNA protection solution	15
Total volume	140

**Table 5:** Sulfite conversion DNA reaction conditions.

Stage	Time	Temperature
Denaturation	5 min	95°C
Annealing	25 min	60°C
Denaturation	5 min	95°C
Annealing	85 min (1 h 25 min)	60°C
Denaturation	5 min	95°C
Annealing	175 min (2 h 55 min)	60°C
Hold	Indefinite	20°C

#### 2.3.6 MassARRAY PCR amplification

PCR amplification for MassARRAY-based DNA methylation detection was performed using the reaction components listed in (**Table 6**), the reaction system in (**Table 7**), and the cycling conditions in (**Table 8**).

**Table 6:** Components of PCR amplification for DNA methylation detection using the MassARRAY system.

Reagent	Final concentration for single reaction	Volume for single reaction
ddH2O	–	6.4 μL
10×PCR Buffer	1 x	1 μL
dNTPs	200 μM	1 μL
PCR Enzyme(5 U/μL)	0.4-0.6 unit/reaction	0.2μL
forward primer(10pmol/μL)	200 nM	0.2 μL
reverse primer(10pmol/μL)	200 nM	0.2 μL
DNA template	–	1 μL
Total Volume:		10 μL

**Table 7:** PCR reaction system.

Reagent	Volume (μl)
Template (20–50 ng/μl)	1
Primer F (10 μM)	2
Primer R (10 μM)	2
dNTP (10 μM)	2
Taq Buffer with MgCl_2_ (10×)	5
Taq enzyme (5 U/μl)	0.5
Water	35.5
Total	50

**Table 8:** PCR reaction conditions.

Procedure	Temperature	Time	Cycles
Pre-denaturation	95°C	5 min	
Denaturation	94°C	30 s	
Annealing	55°C	30 s	35
Extension	72°C	50 s	
Final extension	72°C	8 min	

#### 2.3.7 Pyrosequencing

Pyrosequencing was conducted as follows: All solutions were brought to room temperature prior to use. Annealing buffer (40 μL) containing 0.5 μM sequencing primer was pre-added into each well of the PSQ 96 plate. Streptavidin Sepharose HP beads (GE Healthcare) were vortexed thoroughly to ensure homogeneous suspension. The required volume of Sepharose beads (3 μL per sample) was transferred to an Eppendorf tube, and binding buffer was added to bring the total volume to approximately 40 μL per sample. The bead mixture was then added to the PCR product (40 μL reaction volume) and incubated at room temperature for 5 min with continuous mixing to allow biotin binding. In the Vacuum Prep Workstation, 180 mL each of ultrapure water, 70% ethanol, washing buffer, and denaturation buffer were sequentially added to four separate sample plates. The pump of the Vacuum Prep Workstation was turned on, and the vacuum prep tool was cleaned in ultrapure water for 30 s. The vacuum prep tool was then moved to the PCR plate to capture the Sepharose beads; this operation was completed within 3 min after bead–biotin binding. The PCR plate was lifted to confirm that the majority of beads had been adsorbed onto the vacuum prep tool. The tool was then sequentially placed in 70% ethanol for 5 s, denaturation buffer for 5 s, and washing buffer for 10 s, after which the pump was turned off. The vacuum prep tool was placed into the PSQ 96 plate containing sequencing primers, and the beads were released by gentle shaking. The vacuum prep tool was subsequently cleaned with ultrapure water. The PSQ 96 plate was heated to 80 °C for 2 min and then cooled to room temperature prior to the pyrosequencing reaction. Sequencing and data analysis were performed using PyroMark CpG Software 1.0.11.

#### 2.3.8 Quality control

Sample preprocessing: Genomic DNA was verified by spectrophotometry and agarose gel electrophoresis. Total DNA ≥5 μg, concentration ≥500 ng/μL; no obvious degradation; OD260/280 ratio 1.8–2.0 indicating satisfactory purity.

Pyrosequencing process: Sequencing accuracy was ≥98%. Detection sensitivity down to 10% for low-abundance methylation sites. Quantitative linear range 10%–90% for methylation frequency. Reliable sequencing signal from the first base. Bisulfite conversion efficiency ≥95%; primer binding regions contained at least 4 non-CpG cytosines for conversion monitoring; samples with incomplete conversion were excluded.

Data post-processing: All samples were analyzed in at least two technical replicates, with inter-replicate consistency ≥90% to ensure reproducibility.

#### 2.3.9 RNA extraction and quantitative real-time PCR

Total RNA was extracted from human leukocytes using TRIzol reagent. Samples stored at −80 °C were thawed and mixed at room temperature for at least 15 min. Chloroform (200 μL) was added, and the mixture was gently inverted, incubated at room temperature for 5 min, and centrifuged at 12,000 rpm for 15 min at 4 °C. The upper aqueous phase was carefully transferred to a new tube, mixed with an equal volume of isopropanol, incubated at −20 °C for 60 min, and centrifuged at 12,000 rpm for 15 min at 4 °C. The supernatant was discarded, and the RNA pellet was washed twice with 75% ethanol, followed by centrifugation at 12,000 rpm for 15 min at 4 °C each time. After brief centrifugation, the residual liquid was completely removed, and the pellet was dissolved in RNase-free water. RNA concentration was quantified using a microvolume spectrophotometer, and RNA integrity was verified by agarose gel electrophoresis.First-strand cDNA was synthesized from 1,000 ng of total RNA using the 5X All-In-One RT MasterMix in a final volume of 20 μL. The reaction was incubated at 37 °C for 15 min, followed by 60 °C for 10 min, and enzyme inactivation at 95 °C for 3 min. The resulting cDNA was diluted 1:1 with RNase-free water prior to qPCR analysis. Primers were designed using Primer5 software. β-actin served as the endogenous reference gene. The primer sequences for the target gene SLC6A4 were as follows:

forward, 5’-GGGGCCTTGAAAATTCCAGG-3’; reverse, 5’-GATCCCACCAAAGATTCCGC-3’.

qPCR was performed in a 20 μL reaction mixture containing 10 μL of BlasTaq 2× qPCR Master Mix, 0.5 μL of forward primer, 0.5 μL of reverse primer, 2 μL of diluted cDNA template, and 7 μL of RNase-free water. The thermal cycling conditions consisted of an initial denaturation at 95 °C for 3 min, followed by 40 cycles of denaturation at 95 °C for 15 s and annealing/extension at 60 °C for 1 min. Relative gene expression levels were calculated using the 2^−ΔΔCT^ method, normalized to the internal reference gene.

## 3 Statistical methods

The statistical analysis was conducted using SPSS 26.0. The Shapiro-Wilk test was performed to assess the normality of the data. For normally distributed data, independent-samples t-tests were used to compare the two groups. When data did not follow a normal distribution, the Mann-Whitney U test was utilized. Fisher’s exact test was used to compare categorical data between two groups. Spearman correlation analysis was used to examine the relationships among CpG2 methylation level, SLC6A4 mRNA expression, and total ASLEC score. A *p*-value < 0.05 was considered indicative of statistical significance. A binary logistic regression model was employed to explore the influencing factors of NSSI behaviors in children and adolescents with bipolar depression. The significance level *α* was set at 0.05, and a two-tailed *P*-value < 0.05 was considered statistically significant. Effect sizes were reported for all comparisons: *Cohen’s d* for the continuous variable, *phi (Φ)* for 2×2 categorical comparisons, and *Cramér’s V* for the multi-category educational level variable. *Post hoc* statistical power was calculated using the observed effect size and sample size at a two-tailed *α* = 0.05. The 95% CI for age represents the confidence interval for the mean difference in age. Effect size CIs for categorical variables are not reported due to limited precision at small sample sizes. *Post-hoc* statistical power was calculated using G*Power (version 3.1) based on observed effect sizes at two-tailed *α* = 0.05.

## 4 Results

### 4.1 Comparison of general information

Among the 48 adolescent patients diagnosed with bipolar depression, 33 were assigned to the NSSI group and 15 to the non-NSSI group. The Shapiro-Wilk test was used to check for data normality. The age data in both groups were normally distributed (*p* > 0.05). An independent t-test showed no significant difference in age between the two groups. For categorical variables (gender, educational level, only-child status, family history of mental illness, and disease course), Fisher’s exact test or the chi-square test was used because some expected cell counts were small. The results found no significant differences between the two groups for any of these variables. The results demonstrated that all effect sizes were small, and statistical power was low across comparisons. The confidence interval for the age difference included zero, and tests of categorical variables showed no significant differences between groups. These findings indicate that the two groups were comparable at baseline (**Table 9**).

**Table 9:** Comparison of general data between bipolar depression patients with and without NSSI.

General situation	NSSI group (n = 33)	Non-NSSI group (n = 15)	*t/χ2/Fisher’s*	*p*	Effect size	Power	95%CI
Age	15.39 ± 1.96	16.00 ± 1.73	–1.141	0.260	*d* = 0.36	0.205	[-0.463, 1.675]
Gender				0.509	*Phi* = 0.059	0.087	
Male	8	5					
Female	25	10					
Educational level			1.668	0.434	*V* = 0.186	0.193	
Primary school and below	1	1					
Middle school	15	4					
High school	17	10					
Only child			0.002	0.968	*Phi* = 0.006	0.037	
Yes	13	6					
No	20	9					
Family history of mental illness				1.000	*Phi* = 0.017	0.024	
With	4	2					
Without	29	13					
Disease course				1.000	*Phi* = 0.026	0.275	
First onset	25	11					
Relapse	8	4					

*p*-values from Fisher’s exact test are used when any expected cell count < 5. Effect sizes: *Cohen’s d* for continuous variables (independent t-test); *Cramér’s V* and *phi (Φ)* for categorical variables—95% CI for Cohen’s d computed using normal approximation. The 95% CI for the mean difference in age was obtained directly from SPSS output. *Post hoc* power was calculated using G*Power (version 3.1) based on the observed effect size and sample size, with a two-tailed *α* = 0.05.

### 4.2 Comparison of methylation levels in target segments and SLC6A4 mRNA expression levels of participants with bipolar depression

**Figure 5:**
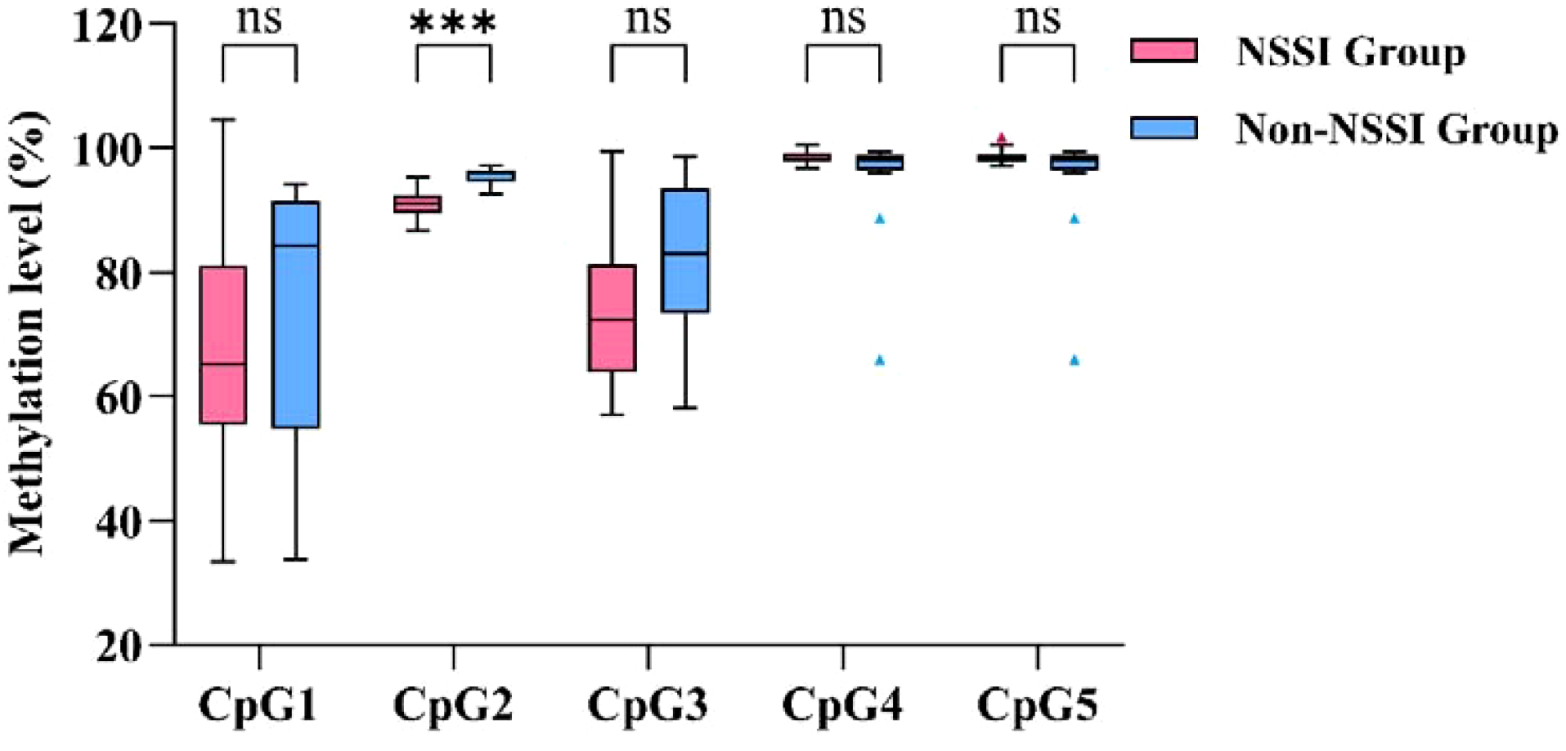
Comparison of methylation levels in target segments participants with bipolar depression.

**Figure 6:**
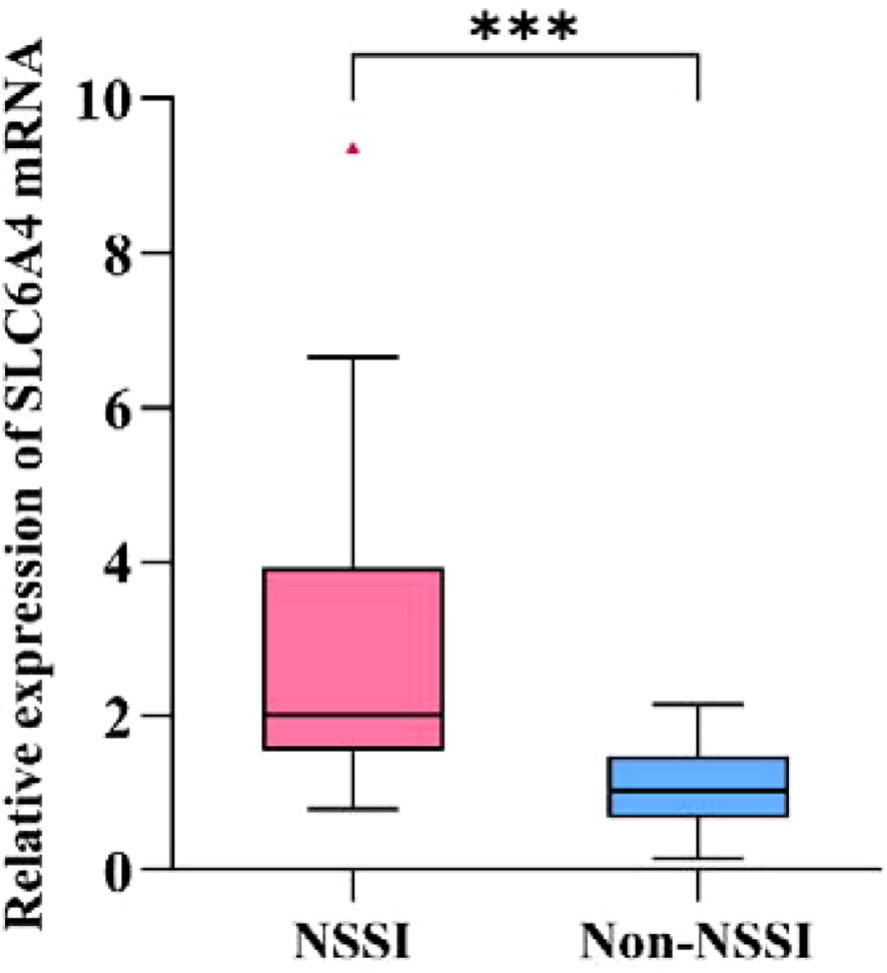
Comparison of SLC6A4 mRNA expression levels of participants with bipolar depression.

SPSS 26.0 was used to compare SLC6A4 promoter methylation and SLC6A4 mRNA expression between the two groups. The Shapiro-Wilk test indicated a non-normal distribution (*p* < 0.05); therefore, the Mann-Whitney U test with Bonferroni correction was applied for group comparisons. The CpG2 site showed a significant difference between groups (*Z* = 5.194, *p* < 0.001, effect size=0.750, 95% CI: [3.50, 5.64]). The NSSI group had lower methylation levels (median = 90.98%, IQR: 89.52–92.42%) than the non-NSSI group (median = 96.14%, IQR: 94.59–96.34%), with a large effect size and high statistical power (0.998). The other methylation sites (CpG1, CpG3, CpG4, and CpG5) showed no significant differences between the two groups. However, these sites had low statistical power, which suggests that insufficient sample size may have led to Type II errors. In particular, CpG3 showed a medium effect size (0.313), and future studies with larger sample sizes are needed to confirm whether real differences exist at these sites. Analysis of SLC6A4 mRNA expression levels showed a significant difference between the two groups (*Z* = 3.915, *p* < 0.001, effect size=0.565, 95% CI: [-2.04, -0.58]). The NSSI group had higher mRNA expression levels (Median=2.00, IQR: 1.54-3.92) compared to the non-NSSI group (Median=1.04, IQR: 0.68-1.49) (**Table 10**, **Figures 5**, **6**).

**Table 10:** Comparison of methylation levels in target segments and SLC6A4 mRNA expression levels of participants with bipolar depression.

Gene	CpG islands	Grouping	M (*P*_25_, *P*_75_)	*Z*	*p*	*Effect size*	*Power*	95%CI
SLC6A4	CpG1	NSSI Group (n = 33)	65.200 (55.460, 81.050)	1.412	0.790	0.204	0.293	9.60 [-5.240,22.440]
		Non-NSSI Group (n = 15)	84.230 (54.840, 91.530)					
	CpG2^*^	NSSI Group (n = 33)	90.980 (89.515, 92.415)	5.194	0.000^*^	0.750	0.998	4.760 [3.500,5.640]
		Non-NSSI Group (n = 15)	96.140 (94.590, 96.340)					
	CpG3	NSSI Group (n = 33)	72.490 (63.815, 81.260)	2.169	0.150	0.313	0.583	9.750 [1.020,17.920]
		Non-NSSI Group (n = 15)	82.970 (73.510, 93.550)					
	CpG4	NSSI Group (n = 33)	98.310 (97.860, 99.185)	1.468	0.710	0.212	0.312	-0.560 [-1.580,0.170]
		Non-NSSI Group (n = 15)	98.090 (96.370, 98.760)					
	CpG5	NSSI Group (n = 33)	98.330 (97.795, 98.905)	1.257	1.000	0.181	0.241	-0.440 [-1.490,0.240]
		Non-NSSI Group (n = 15)	98.090 (96.370,98.760)					
SLC6A4	mRNA	NSSI Group (n = 33)	2.000 (1.543,3.915)	3.915	0.000	0.565	0.987	-1.085 [-2.040,-0.575]
		Non-NSSI Group (n = 15)	1.040 (0.680,1.490)					

*p < 0.001.

### 4.3 Comparison of ASLEC scores between the NSSI group and non-NSSI group in children and adolescents with bipolar depression

The total ASLEC scores between the two groups did not follow a normal distribution; the Mann-Whitney U test was used to analyze group differences. The results showed a statistically significant difference between the two groups (*Z* = 2.215, *p* = 0.027, 95% CI: [-22.00, -1.00]), with higher total ASLEC scores in the NSSI group (median=43.00, IQR: 35.50–55.00) compared to the non-NSSI group (median=35.00, IQR: 21.00–42.00). The moderate effect size (0.32) suggests a meaningful separation between groups. However, the relatively low statistical power (0.55) also indicates that studies with larger sample sizes may be needed to confirm this finding fully (**Table 11**).

**Table 11:** Comparison of ASLEC scores between bipolar depression patients with and without NSSI.

Grouping	M (*P*_25_, *P*_75_)	*Z*	*p*	*Effect size*	*Power*	95%CI
NSSI Group (n = 33)	43.00 (35.50, 55.00)	2.215	0.027^*^	0.32	0.55	-12.000 [-22.00,-1.00]
Non-NSSI Group (n = 15)	35.00 (21.00, 42.00)					

M (P₂₅, P₇₅), median (25th, 75th percentiles); Z, test statistic from the Mann–Whitney U test; Effect size, calculated as r = Z/√N; Power, post-hoc statistical power; 95% CI, 95% confidence interval for the mean difference. *p < 0.001.

### 4.4 Spearman correlations among SLC6A4 mRNA expression, CpG2 methylation, and ASLEC score

**Figure 7:**
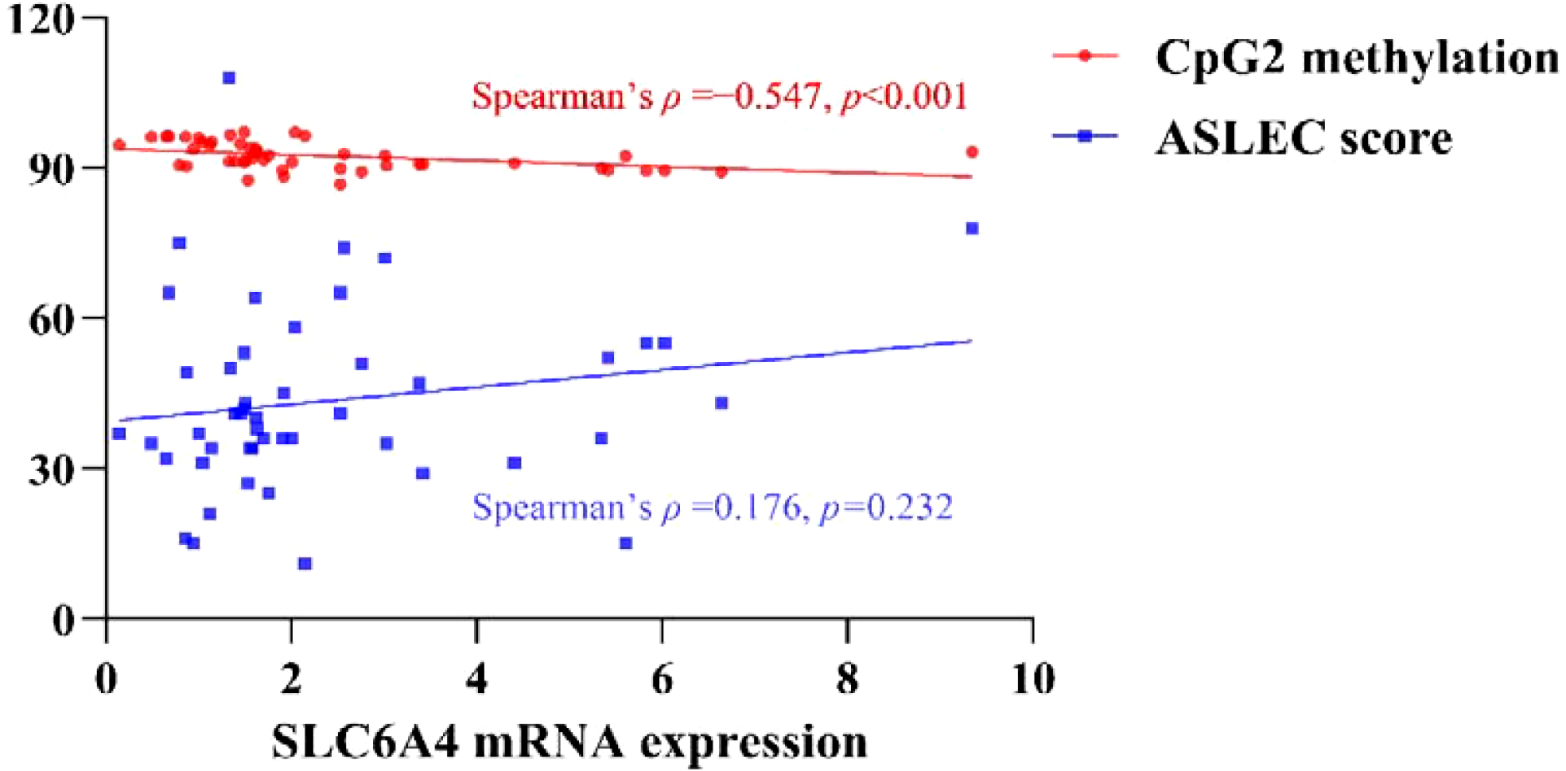
Spearman correlations among SLC6A4 mRNA expression and CpG2 methylation, and ASLEC score.

Spearman correlation analysis revealed a significant negative correlation between SLC6A4 mRNA expression and CpG2 methylation (*ρ* = -0.547, *p* < 0.001, 95% CI: [-0.729, -0.299]), with a *post hoc* power of 0.985, indicating that higher CpG2 methylation was associated with lower SLC6A4 mRNA levels. In contrast, no significant correlation was found between SLC6A4 mRNA expression and ASLEC score (*ρ* = 0.176, *p* = 0.232), nor between ASLEC score and CpG2 methylation (*ρ* = -0.215, *p* = 0.142). The observed effect sizes were small, and the *post hoc* power estimates were low, suggesting that the current sample was underpowered to detect associations of this magnitude (**Table 12**, **Figure 7**).

**Table 12:** Spearman correlations among SLC6A4 mRNA expression, CpG2 methylation, and ASLEC score.

Variable pair	*ρ* (Spearman)	*p*	95% CI *for ρ*	power
SLC6A4 mRNA expression ↔ CpG2 methylation	−0.547	<0 .001^*^	[-0.729,-0.299]	0.985
SLC6A4 mRNA expression ↔ ASLEC score	0.176	0.232	[-0.130,0.466]	0.224
ASLEC score ↔ CpG2 methylation	−0.215	0.142	[-0.465,0.053]	0.314

ρ (Spearman): Spearman correlation coefficient; 95% CI for ρ, 95% confidence interval for the correlation coefficient; Power, post-hoc statistical power. *p < 0.05.

### 4.5 Binary logistic regression analysis of NSSI behavior in children and adolescents with bipolar depression

To explore the factors associated with NSSI in adolescents with bipolar depression, two logistic regression models were constructed. Both models demonstrated acceptable fit (Hosmer-Lemeshow test: *p*=0.853 for Model 1; *p*=0.458 for Model 2).

Model 1 (CpG2 methylation): CpG2 methylation was identified as a significant protective factor against NSSI (OR = 0.131, 95% CI: [0.024, 0.734], *p*=0.021), indicating that higher levels of CpG2 methylation were associated with lower odds of NSSI. In other words, for each unit increase in CpG2 methylation, the odds of NSSI decreased substantially. ASLEC score was not a significant predictor (OR = 1.170, *p*=0.150), and the interaction between CpG2 methylation and ASLEC score was also non-significant (*p*=0.410) (**Table 13**).

**Table 13:** Binary logistic regression analysis of NSSI behavior in children and adolescents with bipolar depression. Mode 1.

Variable	B	SE	Wald χ²	*p*	OR	95% CI
CpG2	-2.030	0.878	5.350	0.021^*^	0.131	[0.024, 0.734]
ASLEC Score	0.157	0.109	2.073	0.150	1.170	[0.945, 1.450]
CpG2 × ASLEC Score	-0.036	0.043	0.679	0.410	0.965	[0.886, 1.051]
Constant	4.390	2.277	3.716	0.054	80.631	—

Variables entered: CpG 2, ASLEC score, and the interaction term of CpG 2 ×ASLEC score; *p < 0.05. OR, odds ratio; CI, confidence interval. Variables were mean-centered prior to analysis. *p* values are two-tailed.

Model 2 (mRNA expression): Higher SLC6A4 mRNA expression emerged as a significant risk factor for NSSI (OR = 10.957, 95% CI: [1.583, 75.867], *p*=0.015), indicating that adolescents with higher SLC6A4 mRNA expression had substantially greater odds of NSSI. The ASLEC score and the interaction term were non-significant (*p* > 0.05) (**Table 14**).

**Table 14:** Binary logistic regression analysis of NSSI behavior in children and adolescents with bipolar depression. Mode 2.

Variable	B	SE	Wald χ²	*p*	OR	95% CI
SLC6A4 mRNA	2.394	0.987	5.880	0.015^*^	10.957	[1.583, 75.867]
ASLEC Score	0.062	0.068	0.843	0.359	1.064	[0.932, 1.214]
SLC6A4 mRNA × ASLEC Score	-0.001	0.054	0.000	0.984	0.999	[0.899, 1.110]
Constant	2.856	1.132	6.368	0.012	17.395	—

Variables entered: SLC6A4 mRNA expression, ASLEC score, and the interaction term of SLC6A4 mRNA expression ×ASLEC score; *p < 0.05. OR, odds ratio; CI, confidence interval. Variables were mean-centered prior to analysis. *p* values are two-tailed. .

## 5 Discussion

This study preliminarily explored the relationship between CpG2 site methylation in the SLC6A4 gene promoter region, SLC6A4 mRNA expression, and NSSI behavior in children and adolescents with bipolar depression. Results showed that the NSSI group exhibited significantly lower CpG2 methylation levels and significantly higher SLC6A4 mRNA expression levels. Logistic regression analysis indicated that both CpG2 hypomethylation and elevated SLC6A4 mRNA expression were independently associated with NSSI behavior.

As a key monoamine neurotransmitter, 5-HT is vital in regulating various physiological and behavioral processes, including appetite, reward processing, sleep, aggression, pain perception, and thermoregulation (17). The serotonin transporter (5-HTT) within this system is the primary target of selective serotonin reuptake inhibitors. Dysfunction of 5-HTT is implicated in the pathogenesis of various mental illnesses, such as mood disorders, schizophrenia, and autism spectrum disorders (18). 5-HTT functions by reuptaking 5-HT from the synaptic cleft, thereby regulating the concentration of 5-HT available for neural signaling. The dysfunction of the 5-HTergic system is associated with NSSI, suicide attempts, and suicidal behaviors. Crowell SE et al. (19) found that the peripheral blood 5-HT levels in adolescents with self-inflicted injury (SII), including suicide attempts and nonsuicidal self-harm, were lower than those in the control group. In animal model experiments (20), rhesus macaques exhibiting self-injurious behavior showed significantly lower 5-HT levels than control animals. NSSI patients demonstrate a therapeutic response to medications that modulate 5-HT function, indicating that the frequency of NSSI episodes can be reduced through the use of selective serotonin reuptake inhibitors (20). Research by Wang et al. (21) has demonstrated that peripheral leukocytes from individuals who experienced childhood physical aggression exhibit distinct methylation patterns in the SLC6A4 promoter region. This finding supports the hypothesis that brain function is linked to peripheral DNA methylation and suggests that SLC6A4 methylation in peripheral tissues may reflect central 5-HT function. They propose that methylation at specific CpG sites within the SLC6A4 gene could serve as a non-invasive biomarker for serotonin synthesis and associated behaviors, such as aggression, that are influenced by altered 5-HT function. Specifically, our findings suggest that changes in DNA methylation within the SLC6A4 promoter may be linked to brain function via central 5-HT levels, and this pathway may be associated with the manifestation of NSSI. However, the current cross-sectional design cannot verify this causal sequence. However, the association between SLC6A4 gene methylation and NSSI behavior in children and adolescents with bipolar disorder has not been investigated in prior research. This preliminary study showed that adolescents with bipolar depression who displayed NSSI behavior had significantly lower methylation levels at a particular CpG site (CpG2) of the SLC6A4 gene compared to those without NSSI. These results imply that NSSI behavior in teenagers with bipolar depression may be biologically correlated with SLC6A4 gene methylation. However, due to the limited sample size, the statistical power to detect differences at the remaining sites is low, which may lead to Type II errors. As a result, we cannot rule out differences in methylation levels between the two groups at these sites (CpG2, CpG3, CpG4, CpG5). Future studies with larger sample sizes are needed to clarify this relationship.

The genomic context affects the relationship between CpG methylation and gene expression. Regardless of whether they are located in a CpG island or not, CpGs less than 2000 bp from the transcription start site (TSS) have a negative correlation with expression (22). Bioinformatic analysis using the UCSC Genome Browser (GRCh38/hg38) revealed that the CpG2 locus (chromosome 17, position 30,236,124) is located within a CpG island in the promoter region of the SLC6A4 gene (23). The CpG2 locus is situated approximately 427 bp upstream of the SLC6A4 transcription start site on the negative strand, within the proximal promoter region. This is consistent with the hypothesis that hypomethylation of CpG sites near the TSS is associated with increased transcription factor binding and elevated gene expression. Previous studies showed that mRNA expression was not significantly correlated with mean methylation across the SLC6A4 promoter CpG island. However, site-level analysis showed that methylation at specific CpG sites, especially those near the transcription start site, was significantly and negatively correlated with mRNA production, with higher methylation at these sites corresponding to decreased transcription (24). A study analyzed 83 CpG sites in the SLC6A4 promoter region and found that methylation at 10 of these sites was negatively correlated with mRNA expression. In contrast, overall methylation showed no significant correlation with mRNA expression (25). This implies that methylation of specific functional CpG sites near transcription factor binding sites, or other epigenetic mechanisms (such as histone modifications), may play a more significant regulatory role than total methylation levels, which may not adequately reflect the state of transcriptional regulation.

To further investigate the functional relevance of this site, JASPAR CORE 2026 (23) was used to predict transcription factor binding sites within the ±200 bp region flanking CpG2 (chr17:30,235,924–30,236,324). Several candidate binding sites were identified, including SP1, SP4, and multiple KLF family members (KLF2, KLF8, KLF9, KLF11, KLF12, and KLF15). SP1, specific protein 1, is one of the earliest discovered transcription factors. It is widely expressed in mammalian cells and plays an important role in gene regulation (26, 27). SP1 binds to GC box sequences in the proximal region of about 27% of human promoters and acts as a transcriptional activator (28, 29). Studies have shown that SP1 is highly expressed in astrocytes, suggesting it may play an important role in their function. Deletion of SP1 in astrocytes alters gene expression, including reduced levels of Toll-like receptor 2 (Tlr2) and complement factor B (Cfb), and increased levels of complement component C1q and C4B-binding protein (C4Bp). These changes can affect neurite outgrowth and synapse formation, leading to neuronal dysfunction (30). Sp4 is a member of the Sp1 transcription factor family and a homolog of the Drosophila buttonhead (btd) gene (31). The same GC-box element is recognized and bound by Sp1 and Sp4 with equal affinity (32). In the central nervous system, Sp1 is specifically expressed in glial cells, while Sp4 is mainly expressed in neurons (33, 34). Studies have found that GC-boxes are significantly enriched in the promoters of schizophrenia-risk genes, indicating that SP4 acts as a regulatory hub, controlling many other risk genes through GC-boxes in the pathogenesis of schizophrenia (35).KLFs are a conserved family of zinc finger transcription factors that bind specific DNA sequences to regulate gene expression. Several members, including KLF4, KLF6, KLF7, KLF9, and KLF10, play roles in neurogenesis and neural differentiation during central nervous system (CNS) development. Beyond development, KLFs also influence neuroinflammation and blood–brain barrier (BBB) integrity. Loss of function in KLF2, KLF4, KLF6, KLF9, and KLF14 has been linked to BBB disruption and increased neuroinflammatory signaling, both of which have been implicated in neuropsychiatric disorders (36). We found that the CpG2 site is located in a critical regulatory region 427bp upstream of the SLC6A4 gene TSS. The methylation status at this site appears to regulate gene expression by affecting the binding of SP1/SP4 and KLF family transcription factors. In the NSSI group, we found lower methylation at the CpG2 site. Whether this alteration influences transcription factor binding remains unclear, and the observed association between CpG2 methylation and increased SLC6A4 mRNA expression was only correlational in the present study. However, given that mRNA expression represents an intermediate phenotype, it remains to be confirmed whether these transcriptional changes correspond to functional differences in serotonin transporter protein levels or activity. Our findings support a potential molecular link between CpG2 hypomethylation and elevated SLC6A4 mRNA expression, although direct causal evidence remains unavailable.

Epigenetic modifications are dynamic and can change in response to environmental factors, including stressful experiences, particularly those occurring early in life, which form the molecular basis of stress-environment interactions. Research by van der Knaap et al. (37) reported that adolescents who experienced stressful life events, traumatic childhood experiences, or perinatal adversity exhibited increased levels of SLC6A4 gene methylation. Similarly, Comtois-Cabana et al. (38) corroborated the influence all treatment and methylation levels at three specific CpG loci within SLC6A4. We found that the total ASLEC score was significantly higher in the NSSI group than in the non-NSSI group, consistent with previous findings and indicating that negative life events are important factors associated with NSSI behavior in adolescents. Adolescents with bipolar depression may lack effective coping strategies when facing life stress and may be more likely to use NSSI as a means of emotional regulation. However, in logistic regression analysis, the ASLEC score was not an independent predictor of NSSI, suggesting that the relationship between stressful life events and NSSI may involve other biological pathways, a possibility that warrants investigation in future studies. Prior research has demonstrated that both childhood and adolescent adversity are associated with altered SLC6A4 methylation (39). In depressed individuals, those with a history of childhood trauma have been shown to exhibit elevated methylation levels, an epigenetic alteration that has been linked to poorer treatment outcomes (40). Interestingly, although the NSSI group experienced more negative life events, ASLEC scores showed no significant correlation with either CpG2 methylation or mRNA expression. Several explanations may account for this observation. First, the ASLEC questionnaire used in this study mainly assesses recent stressful life events and does not include childhood traumatic experiences that may exert lasting programming effects on SLC6A4 methylation. The impact of life events on methylation may be time-dependent, with early-life stress playing a more critical role. Second, altered methylation may represent a correlate or consequence, rather than a cause, of NSSI behavior, though the direction of this association remains undetermined. Third, the limited sample size may have resulted in insufficient statistical power (0.224–0.314). Future studies should enroll larger samples and combine the ASLEC with the Childhood Trauma Questionnaire (CTQ) to more rigorously clarify the distinct epigenetic consequences of environmental exposures across different developmental stages.

The SLC6A4 gene is also implicated in the dysregulation of the HPA axis and cortisol secretion. Research indicates that hypermethylation of the SLC6A4 is linked (41) to elevated concentrations of cortisol and corticosterone in saliva. Extended exposure to severe stress can affect brain regions abundant in glucocorticoid and mineralocorticoid receptors, such as the prefrontal cortex and hippocampus. This may lead to disruptions in HPA axis function (42). The HPA axis mediates responses to chronic stress by releasing cortisol. However, individuals with NSSI exhibit diminished cortisol release in response to stress, reflecting reduced HPA axis reactivity (43). It is possible that stress-related changes in SLC6A4 methylation are associated with alterations in cortisol regulation and related neural pathway function; however, the directionality and causal nature of these relationships cannot be determined from the current cross-sectional design. Our findings may provide potential clues for understanding the biological mechanisms underlying NSSI in adolescents with bipolar depression, and may help identify candidate biological markers associated with NSSI behavior.

Several limitations should be considered when interpreting our findings. First, the sample size was relatively small with unequal group distribution. While the main findings for CpG2 methylation and mRNA expression were statistically significant with large effect sizes, other comparisons (including the remaining four CpG sites and correlations with ASLEC scores) had low statistical power, raising the possibility of Type II errors. The wide confidence intervals in logistic regression models, particularly for mRNA expression in Model 2, indicate instability in effect size estimates. Second, the cross-sectional design precludes determining temporal relationships or causality among SLC6A4 methylation/expression, life stress, and NSSI. A longitudinal investigation is required to clarify whether these epigenetic changes precede or follow the onset of NSSI. Third, we excluded patients with suicidal ideation from this study. Since NSSI and suicidal ideation frequently co-occur in clinical settings, this exclusion criterion may limit our sample’s representativeness of the complex comorbidity patterns commonly seen in clinical practice, necessitating caution in generalizing our findings. Fourth, our study focused exclusively on bipolar disorder patients without including healthy controls. Therefore, we cannot determine whether the observed methylation differences are specific biomarkers of NSSI or reflect broader epigenetic changes associated with bipolar disorder itself. Fifth, recruitment from a single clinical center may limit generalizability to other populations or healthcare settings. Despite controlling for several demographic and clinical variables, residual confounding from unmeasured factors (medication use, comorbidities, or other environmental influences) cannot be ruled out. Sixth, our correlation and regression analyses focused primarily on CpG2, as it was the only site showing significant group differences in univariate analysis. While this stepwise approach is statistically sound and mitigates multiple testing concerns, we cannot rule out the possibility that other CpG sites might show associations in larger samples. Furthermore, a notable limitation of the current study is the reliance on mRNA quantification without direct assessment of serotonin transporter protein levels or functional activity. While SLC6A4 mRNA expression provides a valuable proximal indicator of gene activity, it is not a definitive measure of downstream functional translation. Therefore, the mechanistic implications of our transcript-level findings should be interpreted with caution, and future investigations incorporating direct protein and functional assessments are warranted to strengthen these conclusions. Given these limitations, our findings should be considered exploratory and hypothesis-generating rather than definitive. Future multi-center, adequately powered longitudinal studies that include healthy controls and patients with suicidal ideation are essential to replicate these observations and better understand the complex interplay between SLC6A4 biology, life stress, and non-suicidal self-injury in adolescents with bipolar depression.

## 6 Conclusion

This preliminary study explored the relationship between SLC6A4 promoter CpG2 methylation, mRNA expression, and NSSI behavior in children and adolescents with bipolar depression. Results showed that the NSSI group had lower CpG2 methylation and higher mRNA expression, and that these measures were strongly inversely correlated. Logistic regression indicated that both CpG2 methylation and mRNA expression were independently associated with NSSI. The NSSI group reported higher stressful life event scores, though these did not show a significant moderating effect in the current sample. Given the small sample size and cross-sectional design, these findings should be considered exploratory and require replication in larger longitudinal studies.

## Data Availability

The datasets presented in this article are not readily available due to ethical/privacy concerns. Requests to access the datasets should be directed to zoushaohong@126.com.
